# Pathology of pulmonary tuberculosis: has the tiger changed it’s stripes?

**DOI:** 10.4322/acr.2021.370

**Published:** 2022-04-14

**Authors:** Heena Maulek Desai, Pradeep Vaideeswar, Manish Gaikwad, Gayathri Prashant Amonkar

**Affiliations:** 1 Topiwala National Medical College and BYL Nair Charitable Hospital, Mumbai, Maharastra, India; 2 Seth GS Medical College and KEM Hospital, Mumbai, Maharastra, India

**Keywords:** Tuberculosis, Pulmonary, Bronchopneumonia, Drug resistance

## Abstract

**Background:**

India accounts for the highest number of TB cases globally (almost one-fifth of the global burden and almost two-thirds of the cases in South East Asia. Furthermore, the development of drug resistance of varying levels such as multi-drug resistant TB (MDR-TB), extensively-drug resistance TB (XDR-TB) and total-drug resistant TB (TDR-TB) has been on the increase, and now India also features in the 27 high-MDRTB-burden countries. Almost parallel to these developments, in the last few years, we have been encountering less common morphological forms of pulmonary TB (PTB) at autopsies. With these less common manifestations of the disease, we undertook this study to examine the changing trends in the morphological pattern of pulmonary TB over the recent years.

**Methods:**

In this 3-year retrospective study, adult autopsy cases of PTB (that significantly contributed to the final cause of death) were studied in detail. HIV-positive cases were excluded from the study. The clinical details, gross appearances of the pulmonary lesions, microscopic pattern and Ziehl-Neelsen (ZN) staining were studied. Extrapulmonary involvement and causes of death were documented.

**Results:**

Pulmonary tuberculosis as a cause of death at autopsy was seen in 130 adult patients over 3 years. The age range was between 12 to 70 years. Anti-tuberculous therapy had been administered in 33 of them, but only one patient had taken complete therapy. Dyspnea was the commonest respiratory symptom seen in 51 cases (39.2%). Tuberculous bronchopneumonia was the commonest lesion (45.3%), miliary lesions (including localized miliary) accounted for 26% while fibrocavitary lesions (including the ones not involving apex) were seen in 13% cases. Other morphologies included nodular forms of TB (13%), localized miliary lesions (11.9%), and fibrocavitary lesions, not necessarily involving the apex (11.7% of all fibrocavitary cases), and predominant pleuritis with underlying lung involvement by TB in 1 case. Many cases of TB bronchopneumonia had a bronchocentric pattern of distribution (14.7%). On microscopy, caseating granulomas were seen in 93% cases, only caseation necrosis was seen in 4.6% cases, and necrotizing granulomas with abscess-like reaction in 11.5% cases. ZN staining was positive in 92 cases (70.7%). All the extrapulmonary lesions showed caseating granulomas histologically. The final cause of death was found to be primarily tuberculous in 106 cases (81.5%), whereas in 24 cases (19.5%) pulmonary TB was attributed to the secondary cause of death.

**Conclusion:**

The typical apical involvement of secondary TB was not seen in most of our cases. This could indicate a difference in the morphology and the pattern of lung involvement in recent years. The difference in gross morphology does not affect the pattern of involvement of the lung. In our study, we have observed both; a change in morphology, i.e., more cases of TB bronchopneumonia, and a change in the pattern of involvement like nodular forms, localized miliary forms, and fibrocavitary lesions not necessarily involving the apex. We postulate that this less common manifestation of pulmonary TB is closely related to the development of multi-drug and microbial resistance posing serious medical challenges.

## INTRODUCTION

The concept of a “high burden country” (HBC) for tuberculosis (TB) has been defined by WHO as “a country with a high incidence, prevalence, and mortality from TB.^1^ India is one of the 22 countries in that list, accounting for the highest number of TB cases in the world (almost one-fifth of the global burden, 22.7%) and almost two-thirds of the cases in South East Asia.^1^ This could be due to socio-economic factors, lack of timely and accessible medical therapy, diverse manifestations of the disease, difficulties in identification of the organism, and concomitant HIV infection. Furthermore, the development of drug resistance of varying levels such as multi-drug resistant TB (MDR-TB), extensively-drug resistance TB (XDR-TB) and total-drug resistant TB (TDR-TB) has been on the increase, and now India also features in the 27 high-MDRTB-burden countries.^2^ Almost parallel to these developments, in the last few years, we have been encountering less common morphological forms of pulmonary TB (PTB) at autopsies like tuberculous bronchopneumonia with lower lobe involvement as compared to fibrocavitary TB and acinar nodal foci observed in previous studies. With these less common manifestations of TB, we undertook this study to examine the changing trends in the morphological pattern of pulmonary TB over the recent years.

## MATERIALS AND METHODS

A 3-year retrospective pathological study of PTB was conducted at 2 tertiary-care hospitals in Mumbai. In this period, adult autopsy cases of PTB (that significantly contributed to the final cause of death) were studied in detail. The criteria used for ‘significant pulmonary TB’ was used when pulmonary TB was given as a cause of death and significantly contributor to mortality. Patients with HIV positive status were excluded from the study. The institutional ethics committee had approved the study under the number PG/EC/107/2009. The clinical details noted in all the cases, included the demographic details, total hospital stay, duration and type (constitutional, respiratory and/ or non-respiratory) of symptoms, past history of treated (complete/partial) or untreated TB and presence of associated co-morbid conditions.

**Figure 1:**
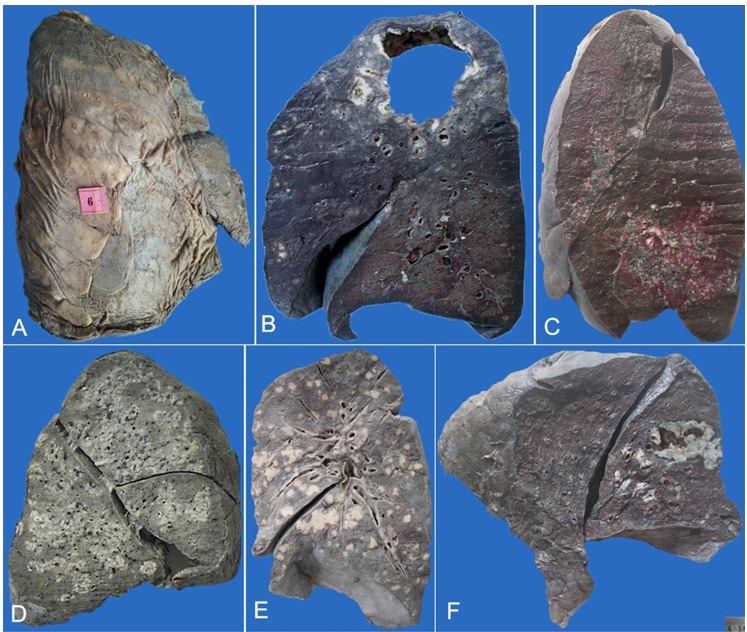
Gross findings of the lungs. **A –** Chronic pleuritis showing thick opaque visceral pleura with adipose tissue infiltration over entire right lung; **B –** Large fibrocavitary lesion in the apical segment of the left lung; **C –** Localized miliary lesions surrounded by congested parenchyma in lingular and basal segments; **D –** Tuberculous bronchopneumonia seen as multi-focal areas of grey-white consolidated areas in the right lung; **E –** Right lung with multiple white nodules scattered all over the parenchyma; **F –** Large solitary nodule with a central area of cavitation.

The gross appearances of the pulmonary lesions (Figure 1) were classified as 1. Fibrocavitary lesions, 2. Tuberculous bronchopneumonia with cavitation, 3. Tuberculous bronchopneumonia without cavitation, 4. Nodular lesions, 5. Miliary lesions and 6. Predominant pleuritis. The fibrocavitary lesions were characterized by a cavity occupying a major part of the lobe / lung (not necessarily the apex) walled off from the lung parenchyma by fibrosis. Areas of tuberculous bronchopneumonia appeared as geographic whitish solidified lesions, at times exhibiting a grape-like appearance. Nodular lesions were those in which the size of the TB lesion was more than 1 cm. Small grey-white pin-headed sized to larger lesions (less than 0.5 cm) constituted miliary tuberculosis. Such lesions could be diffusely distributed in the parenchyma or be localized around the airways. Predominant pleuritis was documented where the pleura showed extensive tuberculous involvement and the underlying lung involvement. Five or more lung sections were studied in all the cases. Particular attention was paid to the type of reaction such as lesions only showing caseation necrosis, well-formed non-caseating / caseating granulomas, poorly formed granulomas composed of collections of macrophages with lymphocytes, necrotizing granulomas or abscess-like reactions; this was noted in particular in the miliary lesions. On microscopic examination, complications were mainly noted like diffuse alveolar damage, organizing pneumonia, hemorrhage, vasculitis, thrombosis and/or infarction. Grossly presence or absence of acute cavitation was noted. Ziehl-Neelsen (ZN) staining was carried out in all cases on scrape cytology and/or on paraffin sections. Extrapulmonary involvement and causes of death were documented.

## RESULTS

In three years, among the 3523 autopsies, there were 130 adult patients (73 men and 57 women) where pulmonary TB was a significant contributor to the cause of death. The age range was between 12 to 70 years, with the maximum number of cases in the third decade. Maximum cases (67%) had a hospital stay less than 24 hours, and the duration of symptoms lasting less than a week was seen in 58.9% of cases. There were nine parturient women. A history of TB was documented in 38 cases (29%). Anti-tuberculous therapy had been administered in 33 of them, but only one patient had taken complete therapy. Associated comorbid conditions included cardiovascular diseases, diabetes mellitus, chronic alcoholism, collagen vascular diseases, renal transplantation, malaria, mental retardation, and muscular dystrophy. Eighty-seven cases (67%) died within 24 hours of hospital stay. Symptoms for 15 days or more had been present in 34 patients (26.2%), while the majority (96 patients, 73.8%) had been symptomatic for less than 15 days. Dyspnea was the commonest respiratory symptom seen in 51 cases (39.2%), and among the non-respiratory symptoms, 24 cases (18.4%) presented with non-specific abdominal pain. Constitutional symptoms such as fever, weight loss, or appetite loss were present in 71 cases (54.6%).

**Figure 2:**
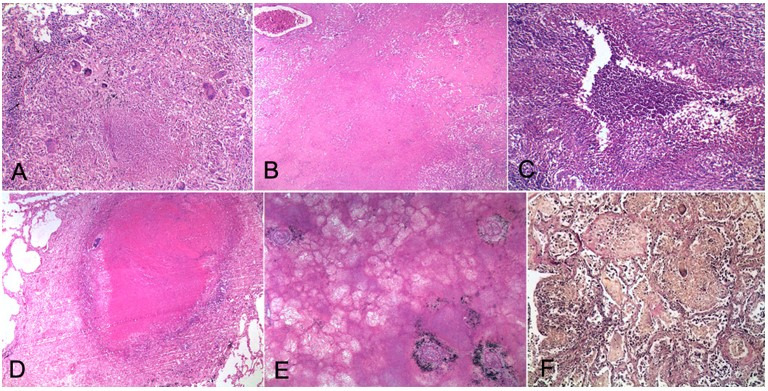
Photomicrographs of the lung. **A –** Caseation necrosis surrounded by epithelioid cells, Langhans giant cells and lymphocytes (H&E, 200X); **B –** A zone composed only of caseation necrosis (H&E, 100X); **C –** Necrotizing granuloma with central abscess-like reaction (H&E, 200X); **D –** Caseation necrosis occluding the lumen of a bronchus. A plate of cartilage is calcified with bluish discoloration (H&E, 100X); **E –** Occlusive arterial lesions with infarction (H&E, 100X); F – Foci of organizing pneumonia with accompanying granulomatous reaction (elastic van Gieson, 200X).

Details of the gross morphology are shown in Table 1, Figure 1AF) and Figure 2AF. Tuberculous bronchopneumonia was the commonest lesion, seen in 59 cases (45.38%) with and without cavitation, miliary lesions accounted for 34 cases (26.15%). In contrast, while nodular lesions, fibrocavitary lesions, and predominant pleuritis were seen in 19 cases (14.62%), 17 cases (13.08%) and 1 case (0.77%) respectively.

**Table 1:** Gross morphology of lung

**Gross morphology**	**Nº of cases**	**%**
TB bronchopneumonia with cavitation	12	9.23%
TB bronchopneumonia without cavitation	47	36.15%
Miliary	34	26.15%
Fibrocavitary	17	13.08%
Nodular	19	14.62%
Predominant pleuritis	1	0.77%

A bronchocentric pattern of distribution was observed in 20.3% of cases of TB bronchopneumonia. Although a diffuse pattern is usually seen with miliary TB, we found localized miliary lesions in 14.7% of miliary TB cases. All the cases had variable lobar distribution. In many cases; especially cases with tuberculous bronchopneumonia and miliary TB, diffuse involvement of the lung was observed, although few cases had a localized miliary pattern of distribution (Figure 1C). Fibrocavitary lesions were seen to involve both apex and other lobes of the lung. Nodular lesions were variable in distribution too. On microscopy, caseating granulomas were seen in 93% cases [more commonly with TB bronchopneumonia (64%)], only caseation necrosis was seen in 4.6% cases [more commonly with fibrocavitary type of TB (15.3%)] and necrotizing granulomas with abscess like reaction were seen in 11.5% cases [more commonly with nodular forms of TB (20.7%)] (Table 2 and Figures 22F).

**Table 2:** Type of reaction

**Type of reaction**	**Nº of cases**
Caseating granuloma	121 (93%)
Caseating necrosis with little reaction	6 (4.6%)
Necrotising inflammation with/ without neutrophils	15 (11.5%)

The correlation of different gross findings with complications like Diffuse alveolar damage (DAD), vasculitis, cavitation, infarction, hemorrhage, and organizing pneumonia are shown in Table 3.

**Table 3:** Correlation of different gross pathologies with complications

	TB broncho pneumonia (n=59)	Miliary TB (n=34)	Nodular TB (n=19)	Fibro-cavitary TB (n=17)	Predominant pleuritis (n=1)
Cavitation	12	-	-	-	-
DAD	4	6*	1	-	-
Vasculitis	16	4	7	5	-
Infarction	1	-	-	-	-
Organising pneumonia	-	1	1	-	-
Hemorrhage	-	1	1	-	-

* (p<0.034, Fischer exact test).

Vasculitis was seen in 55 cases (42%) and was more commonly observed with bronchopneumonia (16 cases). Others included diffuse alveolar hemorrhage (3 cases, 2.3%), diffuse alveolar damage (11 cases, 8.4%), and pulmonary thromboembolism (2 cases, 1.5%). ZN staining was positive in 92 cases (70.7%). Tuberculous dissemination was seen in 52 cases (40%). All these extrapulmonary lesions showed caseating granulomas histologically. The final cause of death was found to be primarily tuberculous in 106 cases (81.5%), whereas in 24 cases (19.5%) pulmonary TB was attributed to the secondary cause of death.

## DISCUSSION

There has been a changing trend in the morphological pattern of pulmonary TB over the years. Between 1948 - 1978, tuberculous cavitation was the most common finding seen in 126 out of 168 cases (75%), followed by advanced pulmonary TB without cavitation in 25 cases (15%).^3^ Ubaidullaev et al.,^4^ in their 1998 autopsy study, also found fibrocavitary TB (55.9%) to be the commonest type. They even concluded that mortality related to TB occurred more commonly as a complication of fibrocavitary TB (35.2%) compared to hematogenous spread. ^[4]^ Later such cases reduced to almost half in 2009.^5^

In the year 2006, Theegarten et al.^6^ studied 55 (1.39% cases of TB and found acinar nodal foci as the commonest lesions seen in 16 cases (29.1%), caseous pneumonia and miliary TB were found in only 8 (14.5%) cases. In contrast, cavitary lesions were seen in 15 cases (27.3%). Another study by Yamamoto et al.^7^ in 2009 studied 18 cases of active TB during autopsy and found caseous pneumonia in 6 cases (33%), and encapsulated nodular lesions were seen in 7 cases (39%), whereas, in an Indian study done in 2011, cavitary lesions were commoner.^8^ Our study showed a predominant bronchocentric pattern of spread and consequent tuberculous bronchopneumonia (45.3%) as the most common manifestation in 130 cases of pulmonary TB, where TB significantly contributed to the final cause of death. A recent Indian study by Gupta et al.^9^ found TB bronchopneumonia to be the most common pattern of lung involvement (27.5%), closely followed by miliary TB (25%). Fibrocavitary TB was found in only 12.5% of their cases, indicating a sharp decline in the fibrocavitary pattern seen in our study. Thus, although a geographical variation was seen in findings from Northern and Western India in 2011, similar findings have now been noted in both these regions in 2016.^8^^,^^9^ TB bronchopneumonia can be a cause of sudden death in TB.^10^ Nowadays, we are experiencing more bronchocentric lesions in TB rather than peripheral lesions. There are very occasional case reports in the literature on bronchocentric distribution of TB.^11^

Although atypical patterns of lung involvement were seen on gross examination of the lung, microscopy showed maximum cases (93%) demonstrating caseating granulomas in both the pulmonary and extrapulmonary lesions. This could be due to the fact that we have excluded patients with the diagnosis of HIV infection in our study, which usually shows atypical microscopic patterns like florid caseation necrosis without any classical granulomas.^12^

The correlation of different gross pathologies, specifically with complications like DAD, vasculitis, cavitation, infarction, organizing pneumonia, and hemorrhage, have been highlighted in Table 3. TB vasculitis was seen in 32 cases (24.6%), suggesting a very high percentage of vasculitis in pulmonary TB. We found 11 cases (8.4%) with DAD. In cases of TB, there is an accumulation of inflammatory cells within the alveolar spaces causing the release of enzymes and cytokines, which cause damage to the alveolar basement membrane leading to the formation of hyaline membranes.^13^ Sharma et al.^14^ in their study have concluded that in TB patients, prolonged course of illness and/or decreased lymphocyte count and/or raised values of alanine transaminase (ALT), low platelet counts and serum albumin levels are all independent risk factors for the development of DAD. Out of all morphological patterns of TB, DAD has been reported with miliary TB only and is associated with a high mortality rate. The largest study done so far was by Deng et al.^13^ between the years 2006-2010, where 85 cases of miliary TB and DAD were found. In our study, DAD was more commonly seen with miliary TB. This was found to be statistically significant (p<0.0354) (Fischer exact test).

Another perplexing fact is that only 38 cases (29%) had a history of tuberculosis, of which only 1 case had taken complete treatment. This could reflect the atypical morphological patterns of pulmonary TB in our cases. One more possibility could be the development of resistance to anti-tuberculous drugs as maximum cases had taken incomplete treatment.

The most common associated comorbidity was diabetes mellitus, seen in 17 cases (13%). Diabetes mellitus has always been a commonly associated co-morbid condition with TB over the years. Feleke et al.^15^ in 1997, studied 1352 people with diabetes mellitus and found 78 cases of TB with a prevalence as high as 5.8%, indicating an increased relative risk of developing TB in diabetics. Restrepo^16^ has mentioned that cavitary lesions in the lung are more common in people with diabetics than non-diabetic cases with TB. In our study, in diabetics with PTB; we found more cases of TB bronchopneumonia (58.8%) than fibrocavitary lesions (11.7%).

In our experience, 9 cases (6.9%) were females in the post-partum period. In early pregnancy, non-specific symptoms like weakness and loss of appetite are usually attributed to the pregnant state. Also, X-rays being contra-indicated in pregnant women because of risk to the fetus, the diagnosis of TB is further delayed. The maternal deaths due to TB was 6.6 deaths per 100 infected mothers in 1992 and still constitutes 6%-15% of all maternal mortality deaths in 2012.^17^^,^^18^

The other comorbidities seen with TB cases included Hepatitis C infection, human immunodeficiency virus (HIV) infection, cardiovascular diseases, cancer, and chronic obstructive pulmonary disease.^19^ In our study, we have not included cases with concomitant HIV infection, which would have further increased the prevalence rate of TB.

The typical apical involvement of secondary TB was not seen in most of our cases. This could indicate a difference in the morphology and the pattern of lung involvement in recent years. An autopsy-based article by Blacklock^20^ was published in 1932 when there were no drugs to treat tuberculosis; so, one could not ascribe, at least among children, the high incidence of miliary and bronchopneumonia to the emergence of drug-resistant microorganisms. It seems more appropriate to ascribe these findings to the complete absence, at that time, of effective drug treatment for the disease, so as to increase the severity of the lesions and, consequently, the risk of death. Now, with the advent of microbial resistance to drugs, we are getting morphologies similar to those seen in the pre-antibiotic age.

We postulate that this new emerging morphological pattern of pulmonary TB is closely related to the development of multi-drug and microbial resistance posing serious medical challenges.

Since large autopsy studies of Pulmonary TB in India are scarce and the microbial resistance pattern is unavailable in the majority of cases, this study would help in the future to study this new pattern of involvement of the lung in TB.

## LIMITATIONS OF OUR STUDY

Since this was a retrospective observational analysis of pulmonary features, we could offer no comment on the pathogenetic mechanisms of the mycobacteria-host interaction, and there was no scope of performing immunological markers either.
